# The Crystal Structure of the Core Domain of a Cellulose Induced Protein (Cip1) from *Hypocrea jecorina*, at 1.5 Å Resolution

**DOI:** 10.1371/journal.pone.0070562

**Published:** 2013-09-05

**Authors:** Frida Jacobson, Saeid Karkehabadi, Henrik Hansson, Frits Goedegebuur, Louise Wallace, Colin Mitchinson, Kathleen Piens, Ingeborg Stals, Mats Sandgren

**Affiliations:** 1 Department of Molecular Biology, Swedish University of Agricultural Sciences, Uppsala, Sweden; 2 DuPont, Industrial Biosciences, Leiden, The Netherlands; 3 DuPont, Industrial Biosciences, Palo Alto, California, United States of America; 4 Department of Biochemistry, Physiology and Microbiology, Ghent University, Ghent, Belgium; 5 Faculty of Applied Bioscience Engineering, University College Ghent and Ghent University, Ghent, Belgium; University of Oulu, Finland

## Abstract

In an effort to characterise the whole transcriptome of the fungus *Hypocrea jecorina*, cDNA clones of this fungus were identified that encode for previously unknown proteins that are likely to function in biomass degradation. One of these newly identified proteins, found to be co-regulated with the major *H. jecorina* cellulases, is a protein that was denoted Cellulose induced protein 1 (Cip1). This protein consists of a glycoside hydrolase family 1 carbohydrate binding module connected *via* a linker region to a domain with yet unknown function. After cloning and expression of Cip1 in *H. jecorina*, the protein was purified and biochemically characterised with the aim of determining a potential enzymatic activity for the novel protein. No hydrolytic activity against any of the tested plant cell wall components was found. The proteolytic core domain of Cip1 was then crystallised, and the three-dimensional structure of this was determined to 1.5 Å resolution utilising sulphur single-wavelength anomalous dispersion phasing (sulphor-SAD). A calcium ion binding site was identified in a sequence conserved region of Cip1 and is also seen in other proteins with the same general fold as Cip1, such as many carbohydrate binding modules. The presence of this ion was found to have a structural role. The Cip1 structure was analysed and a structural homology search was performed to identify structurally related proteins. The two published structures with highest overall structural similarity to Cip1 found were two poly-lyases: CsGL, a glucuronan lyase from *H. jecorina* and vAL-1, an alginate lyase from the *Chlorella* virus. This indicates that Cip1 may be a lyase. However, initial trials did not detect significant lyase activity for Cip1. Cip1 is the first structure to be solved of the 23 currently known Cip1 sequential homologs (with a sequence identity cut-off of 25%), including both bacterial and fungal members.

## Introduction

The filamentous soft-rot fungus *Hypocrea jecorina* (previously *Trichoderma reesei*) [1] secretes large quantities of carbohydrate degrading enzymes that act synergistically to degrade cellulose and related plant biomass components. The cellulolytic and hemicellulolytic machinery of this organism has been studied intensively over the past fifty years as a model system. Recent focus has been on its use in the conversion of lignocellulose biomass feed stocks into fermentable sugars to be used in bio-fuel production. The enzymes in the cellulolytic machinery of *H. jecorina*, as well as carbohydrate degrading enzymes from other organisms, are classified in different glycoside hydrolase (GH) families in accordance with the classification system of Henrissat and co-workers [2], [3]. The classification is based on sequence similarities between the proteins, and consequent conservation of fold and stereochemical outcome of the catalyzed reaction, i.e. inversion (single displacement) or retention (double displacement) of the anomeric configuration at the scissile bond [4], [5].

The gene products of *H. jecorina* include at least four endoglucanases (EG, EC 3.2.1.4), Cel5A, Cel7B, Cel12A and Cel45A (previously known as EG II, EG I, EG III and EG V, respectively), two exoglucanases or cellobiohydrolases (CBH, EC 3.2.1.91), Cel6A and Cel7A (previously known as CBH II and CBH I, respectively), and at least two members of GH family 61, now thought to be lytic polysaccharide mono-oxygenases, GH family 61A and GH family 61B (previously known as EGIV and EGVII, respectively) [6].

In an ongoing effort to further characterise the *H. jecorina* genome, over 5100 random cDNA clones were sequenced [6]. Among these sequences, 12 were identified that encode for previously unknown proteins that are likely to function in biomass degradation. The analysis was based on sequential similarity but co-regulated proteins were also considered. One of these newly identified proteins that were found to be co-regulated with the major *H. jecorina* cellulases was a protein that was denoted Cellulose induced protein 1 (Cip1).

In this paper we present the work to identify, clone and express the *H. jecorina cip1* gene, biochemical characterization of the protein, and the solution of its three-dimensional structure by x-ray crystallography. Cip1 is the first structure to be solved of the 23 currently known Cip1 homologues (extracted from protein BLAST search with a sequence identity cut-off of 25%), including both bacterial and fungal members. We analyse some important features of the Cip1 structure, including its similarities to other carbohydrate active proteins, and discuss the relevance of these observations to our ongoing research to better characterise the activities and functions of the lignocellulosic degrading machinery of *H. jecorina*.

## Results

### Identification of the cip1 gene

From an extensive investigation of a large cDNA library of *H. jecorina* QM6a, a new gene was identified and named “cellulose induced protein 1” (Cip1). This gene was also cloned and transformed back into *H. jecorina* as described in the Materials and Methods section. The *cip1* gene sequence (UniProt ID: Q7Z9M9) consists of an N-terminal signal peptide (19 residues), a core domain (218 residues), a linker region (40–45 residues) and a C-terminal carbohydrate binding module (CBM) family 1 sequence (35–40 residues). A BLAST protein sequence similarity search, using the BLAST server at NCBI (http://blast.ncbi.nlm.nih.gov), was performed to identify homologous protein sequences. This BLAST homology sequence search revealed the existence of a total of 23 protein sequences from diverse organisms as fungi, actinomycetes, chloroflexi and proteobacteria. A total of 14 bacterial sequences were found (using a sequence similarity cut-off of 25%), of which at least 12 contain an N-terminal CBM family 2 domain, including the *H. aurantiacus* homolog that also contains a C-terminal chitinase-like domain. Of the 14 bacterial homologs, eleven are actinomycetes, two are chloroflexi and one is proteobacteria. From the nine published fungal Cip1 homologs, only the *Chaetomium globosum* homolog showed a C-terminal CBM domain, though of family 1 and not of family 2 as seen in the other homologues – 65% similarity was found between the Cip1 core domain and this uncharacterised putative protein (Q2GNC6_CHAGB).

**Figure 1:**
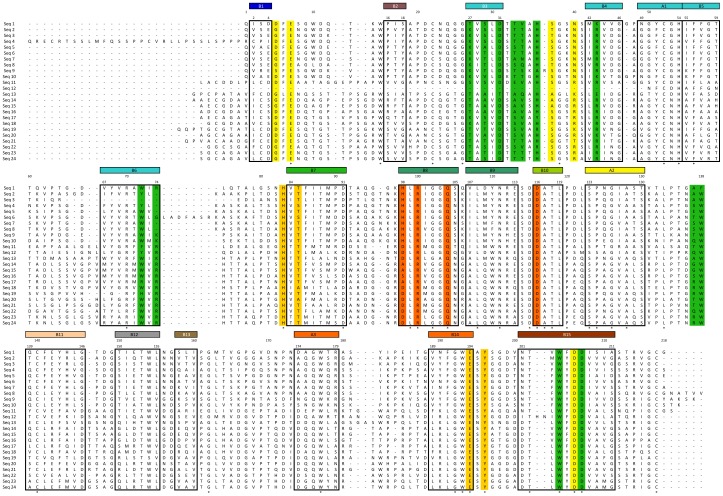
Sequence alignment of Cip1 homologs. Sequence alignment of *H. jecorina* Cip1 amino acid sequence with all publically available protein sequences with a BLAST identity percentage of at least 25%. Sequences 1–10 are fungal sequences and sequences 11–24 are from bacteria. The residues marked in green are located in the “grip” region (fig. 8), the residues marked in bright orange are plausible active site residues in the cleft of the structure, the light orange residues are located together on one side of the cleft interacting with an ethylene glycol molecule in the Cip1 structure and the residues marked in yellow interact with a calcium ion in the “grip” region of Cip1. The secondary structure is marked with boxes and each element coloured according to the rainbow colouring in the related topology diagram (fig. 3). The shown aligned sequences (EMBL Genbank access numbers indicated in parentheses) are: seq. 1, *Hypocrea jecorina* Cip1 (AAP57751); seq. 2, *Pyrenophora teres f teres* 0–1 (EFQ89497); seq. 3, *Pyrenophora tritici repentis* (XP_001937765); seq. 4, *Chaetomium globosum* (XP_001228455); seq. 5, *Chaetomium globosum* (XP_001222955); seq. 6, *Phaeosphaeria nodorum* SN15 (XP_001790983); seq. 7, *Podospora anserina* S mat+ (XP_001906367); seq. 8, *Magnaporthe oryzae* 70-15 (XP_365869); seq. 9, *Nectria haematococca* mpIV (XP_003039679); seq. 10, *Gibberella zeae* PH-1 (XP_386642); seq. 11, *Haliangium ochraceum* DSM 14365 (YP_003266142); seq. 12, *Herpetosiphon aurantiacus* ATCC 23779 (YP_001545140); seq. 13, *Catenulispora acidiphila* DSM 44928 (YP_003114993); seq. 14, *Streptomyces coelicolor* A3(2) (NP_629910); seq. 15, *Streptomyces lividans* TK24 (ZP_05523220); seq. 16, *Streptomyces sp*. ACTE (ZP_06272077); seq. 17, *Streptomyces sviceus* ATCC 29083 (ZP_06915571); seq. 18, *Streptomyces sp*. e14 (ZP_06711846); seq.19, *Actinosynnemma mirum* DSM 43827 (YP_003101274); seq. 20, *Amycolatopsis mediterranei* U32 (YP_003767350); seq. 21, *Streptomyces violaceusniger* Tu 4113 (ZP_07602526); seq. 22, *Cellulomonas flavigena* DSM 20109 (YP_003638201); seq. 23, *Micromonospora aurantiaca* ATCC 27029 (YP_003835070); seq. 24, *Micromonospora sp.* L5 (YP_004081730).

Comparison of core domain sequences of the homologs to the core domain sequence of Cip1 from *H. jecorina* showed moderate similarity to bacterial homologous sequences (38%–43%) with no significant difference due to bacterial origin (actinomycete, chloroflexi or proteobacteria). Comparison of the core domain sequence of Cip1 from *H. jecorina* to nine fungal homologous core domain sequences revealed significantly higher similarity (58%–67%). An alignment of all Cip1 homologous sequences is shown in Figure 1. The pairwise amino acid sequence identity percentages between all known Cip1 homologues are shown in Figure S1 (supplementary material).

Foreman *et al.* [6] did show that, among different strains of *H. jecorina* with varying cellulase-producing capabilities and under various growth conditions, the regulation of the *cip1* gene at mRNA-level is indistinguishable from the expression levels of the fungal cellulases and, in particular, from its cellobiohydrolase *Cel7a*. The co-regulation of Cip1 with the other cellulase components in the fungus, and the fact that it contains a CBM, points towards a role (catalytic or carbohydrate binding) for Cip1 in the degradation of complex cellulose substrates. Determining the structure and testing the Cip1 protein under different conditions should thus be helpful in the identification of its biological properties.

### Biochemical characterisation

Cip1 protein, intact with both catalytic core domain and CBM, was assayed for hydrolytic activity on a range of carbohydrate substrates. After extensive purification Cip1 did not reveal any activity in: 1) overnight assays against the chromogenic substrates 2-chloro-4-nitrophenyl-β-D-glucoside (CNPG), 2-chloro-4-nitrophenyl-β-D-cellobioside (CNPG2) and 2-chloro-4-nitrophenyl-β-D-lactooside (CNP-Lac); 2) against cellopentaose and 3. in gel diffusion assays against cellulose and hemicellulose substrates (data not shown). Thus, no β-glucosidase or cellulase activity could be detected for Cip1. Also, Cip1 did not show any synergistic effect with cellobiohydrolase Cel7A on crystalline cellulose (cotton linters), nor on amorphous cellulose (phosphoric acid swollen cellulose, data not shown).

Binding of Cip1 to soluble polysaccharides, both as intact protein and as the proteolytic core domain only, was explored using affinity gel electrophoresis. No change in migration time was observed for the Cip1 core domain under the conditions used (see Materials and Methods section). For instance, after removal of the CBM1, no adsorption onto avicel cellulose was observed with the Cip1 core domain. Interestingly, the migration of intact Cip1 was delayed in xyloglucan-containing native gels. This retention is most likely due to the presence of the CBM1 module in intact Cip1, as a similar observation was made for intact Cel7A compared to the Cel7A core domain (data not shown). Thus, the family 1 CBM is also able to accommodate the side chains of xyloglucan, as was previously observed for the CBMs from family 30 and 44 [7].

Since three-dimensional protein structure is more conserved than amino acid sequence, we decided to determine the crystal structure of Cip1 to enable the search for structural homologs and, thereby, for a potential role for this protein in biomass degradation. In the discussion section a detailed analysis of the Cip1 structure is showing that the closest structural homologs found function as lyases. Cip1 was thus tested for lyase activity with the substrate glucuronan, but only very low catalytic activity was seen and the signal-to-noise ratio was low, making these measurements uncertain. The addition of metal ions (divalent Fe, Ni, Zn and Mg) to the protein solution prior the activity measurements increased the potential activity signal, but the experimental values were still too low for the detected activity to be considered as convincing.

### Overall structure analysis and validation

The proteolytic core part of Cip1 was crystallised and the structure determined with sulphur-SAD to a final resolution of 1.5 Å. The Cip1 structure model contains 1994 non-hydrogen atoms belonging to 218 amino acid residues, one N-acetylglucosamine (NAG) residue (from the glycosylation of Asn156), one calcium ion, one PEG molecule, eight ethylene glycol molecules and 200 water molecules. There is a disulfide bond between Cys22 and Cys52, although probably partially destroyed by radiation damage during x-ray data collection. A second disulfide bond may exist between Cys140 and Cys217, but if so, the radiation damage was too severe for the cysteines to be modelled in conformations allowing for S-S bonding. The side chains of 17 residues in the structure show alternate conformations: Ser8, Thr13, Ser18, Cys22, Cys52, Val62, Val67, Ser81, His98, Asp116, Glu142, Val165, Ser181, Val200, Val203 and Ser212. The final structure model has a crystallographic R-factor of 19.1% and an R-free value of 21.7% for the resolution range of 45.6 - 1.5 Å. Further structure model and refinement statistics are provided in Table 1. The final σ_A_-weighted 2|Fo|-|Fc| electron density map shows continuous electron density for all main chain atoms of the structure. In the Ramachandran plot [8], none of the non-glycine residues in the structure are outliers by the stringent core definition of Kleywegt and Jones [9], and other geometric parameters show only small deviations from ideal values. The first visible amino acid in the electron density at the N-terminus of Cip1 is a pyro-glutamate (PCA) residue. This is residue 20 of the deposited amino acid sequence of Cip1 (UniProt ref: **Q7Z9M9**), but is predicted to be the first residue in the mature form of the protein, following removal of the signal peptide. Therefore, we have decided to start the numbering of the amino acids in the Cip1 structure from glutamine 20 of the deposited amino acid sequence. Thus, Gln20 in the deposited amino acid sequence of Cip1 is denoted PCA1 in the presented and deposited Cip1 protein structure.

**Table 1:** Diffraction data, processing, phasing and structure refinement statistics.

*H. jecorina* Cip1 data set	S-SAD	Merged dataset
***A. Data collection and processing***		
Beamline^a^	BM14	ID23-1
Detector	CCD 225	CCD 225
Wavelength (Å)	1.771	0.979
Oscillation range (^o^)	1.0	0.5/2.0
Number of images	720	360/90
Angle of total revolution (^o^)	720	180/180
Space group	P212121	P212121
Cell parameters (a, b, c: Å)	57.9, 60.0, 77.5	55.4, 57.5, 74.6
Resolution range (Å)	20-2.0	10-1.5
Resolution range outer shell	2.03-2.0	1.53-1.50
No. of observed reflections	859917	2745135
No. of unique reflections	18867	38981
Average multiplicity^b^	18.2 (17.5)	6.2 (6.4)
Completeness (%)	100 (100)	99.9 (99.9)
I/σ(I)	46.05 (9.5)	20.7 (2.6)
***B. Phasing***		
Resolution cut-off (Å)	20-2.0	
No. of sites	13	
R-anomalous	0.02	
C.C-anomalous	34.5	
Overall phasing power	1.36	
Overall figure of merit		
Acentric reflections	0.406	
Centric reflections	0.116	
***C. Refinement and final structure model***		
PDB access code		3ZYP
Resolution used in refinement (Å)		46-1.5
Reflections in: total and test set		36753; 1951
R and R_free_ factor (%)		19.1; 21.7
Protein molecules in AU		1
Residues in protein		218
Non-hydrogen protein atoms		1698
Waters		200
Residues with dual conformations		18
Calcium atoms		1
PEG molecule		1
Ethylene glycol molecules		8
N-glycosylation molecules		1
Average atomic B-factor (Å^2^):		
overall		17.9
protein		14.5
water		27.4
calcium		8.7
RMSD bond lengths from ideal (Å)		0.009
RMSD bond angles from ideal (°)		1.31
Ramachandran outliers (%)		0.0

a Beamlines at the European Synchrotron Radiation Facility (ESRF), Grenoble, France.

b Numbers in parentheses are for the highest resolution bins.

The table values were calculated with O [41], [46], Refmac5 [37], CNS [47], MOLEMAN [48], and LSQMAN [49]. Calculated using the strict boundary Ramachandran definition given by Kleywegt and Jones [9].

### Protein fold

**Figure 2:**
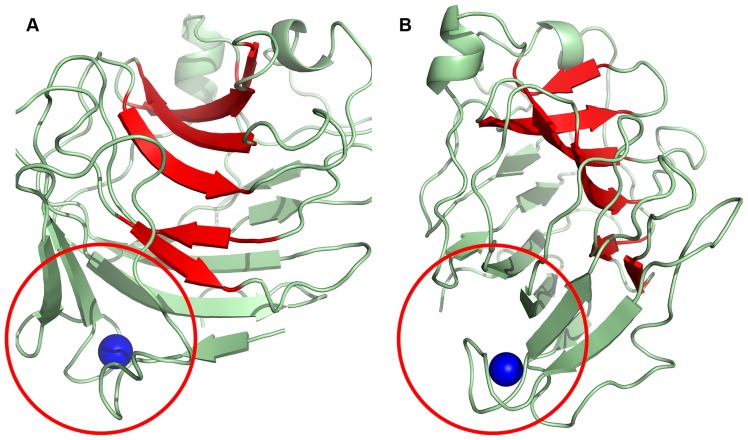
Overall view of Cip1. Overall view of *Hypocrea jecorina* Cip1 showing the structure in A) front view and B) side view. The β-strands that make up the bottom of the cleft (β-sheet B) are coloured in red, forming a β-sandwich together with β-sheet A (green). A red circle surrounds the “grip” motif where a calcium ion is also found (blue).

The Cip1 core domain structure is best classified as having a β-sandwich jelly-roll fold. It comprises a compact, globular, single domain built up of two anti-parallel β-sheets, named A and B, which pack on top of one another (Figure 2). The two β-sheets consist of a total of 15 β-strands, eight in β-sheet A and seven in β-sheet B. One of these β-sheets (B) forms the floor of a large cleft and in the lower part of the molecule there is a “grip”-like motif (Figure 2a) where part of the other β-sheet (A) forms the “palm” and a protruding loop forms the “bent fingers”(Figure 2b). This loop binds the calcium ion that can be seen in other structures, found to be structurally homologous to Cip1, both catalytic domains and CBMs. However, this calcium ion cannot be viewed as a criterion for either activity or sugar binding but rather as having a stabilising effect on the β-jelly-roll fold. The effect of calcium on the stability of CBM proteins has been thoroughly examined by Roske *et al.* [10].

**Figure 3:**
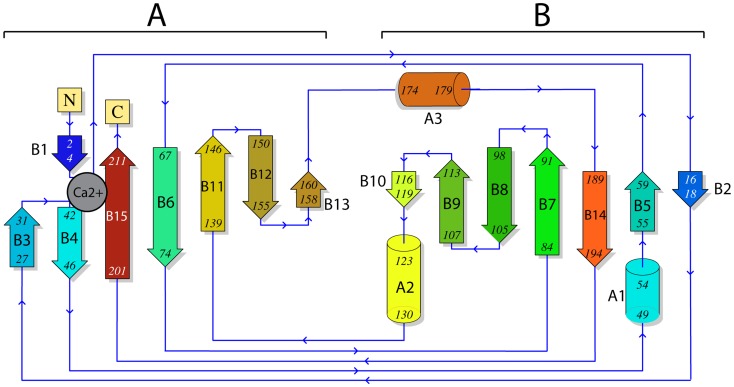
Topology diagram of Cip1. Secondary structure of *Hypocrea jecorina* Cip1 coloured in rainbow from N-terminal blue to C-terminal red. The concave active site cleft β-sheet is on the right in the topology diagram (β-sheet B). The “grip” motif is on the left, in part consisting of the outer convex β-sheet “palm” (β-sheet A) and the “bent fingers” formed by the loop of residues 32–41. The calcium ion is depicted in grey and coordinates residues from both the N-terminal and C-terminal as well as from the loop in the grip motif, thereby stabilizing the structure in that area.

In addition to the 15 β-strands in the Cip1 structure, three α-helices are present. The secondary-structure elements of the Cip1 structure were divided into α- and β-elements, then numbered according to the order of their occurrence in the amino acid sequence of the protein and rainbow coloured (Figure 3). The Cip1 structure is relatively compact without any extended loop regions, and with overall dimensions of approximately 40 Å×38 Å×37 Å.

### The calcium binding site

After solving the structure, inspection of the electron density revealed the possible presence of a metal atom bound in the structure. This metal gave rise to the strongest peak in the anomalous difference Fourier electron density map, 24σ compared to 10σ for the second strongest site, which corresponds to a sulphur atom of a cysteine residue in the structure. The metal binding site is situated on the opposite side of the plausible active site cleft, held by the loop in the “grip” motif described above as well as the N- and C-terminal regions of the Cip1 core domain. The nature of this potential metal atom was unknown, thus several atoms were modelled during the refinement. A calcium atom was found to provide the best fit with regards to both B factor and metal coordination geometry. To further confirm the identity of the metal bound to the protein, a sample of Cip1 was characterised by particle-induced X-ray emission (PIXE). The PIXE spectrum (data not shown) unambiguously identified the presence of one calcium atom bound for each Cip1 molecule in solution.

Whether calcium has any role in the substrate binding or catalytic ability of Cip1 or not remains unclear since the exact function of the protein is not known. However, calcium has a clear structural role in Cip1 due to its critical position in the structure of the protein. The contribution of calcium to the stability of protein structures has been an object for extensive study [11]. The effect of calcium on the stability of β-jelly-roll fold CBM structures has been thoroughly examined by Roske *et al.* [10]. To establish the importance of calcium for the stability of Cip1, thermal denaturation experiments were performed to study stability and reversibility of Cip1 in the absence and presence of ethylene-diamine-tetra-acetate (EDTA), a metal ion chelator.

**Figure 4:**
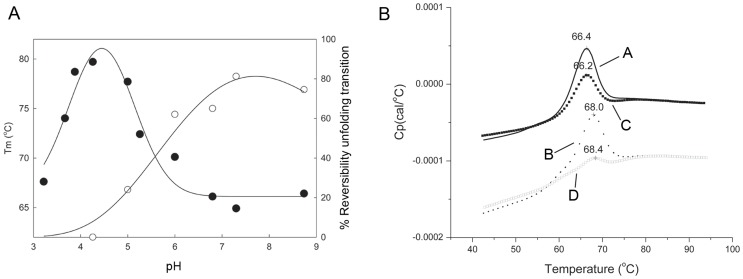
Thermal unfolding of Cip1. Panel **A** shows two different curves, one showing pH dependence of the thermal unfolding midpoints (Tm; •) and the other showing pH dependence of the reversibility of the amplitude of unfolding for Cip1 (o). The differential scanning calorimetry profiles were collected over pH range of 3.2-to-8.8. The data was collected from 30–90°C at a scan rate of 200°C/hr using the VP-Cap DSC (MicroCal, Inc. Northampton, MA). The reversibility of the unfolding amplitudes was calculated using Peakfit v.4.12 (Seasolve Software, Inc, MA). The solid lines are to guide the eye. Panel **B** shows the thermal unfolding profiles for Cip1 at pH 6.8 in the absence (A) and presence (B) of 5 mM ethylene-diamine-tetra-acetate (EDTA). Rescans of the thermally unfolded samples in the absence (C) and presence (D) of EDTA are also shown. All scans were performed at 200°C/hr over a temperature range of 30–90°C using Auto-Cap DSC (MicroCal, Northampton, MA).

To investigate how pH affects the protein thermal stability and folding reversibility, thermal denaturation experiments by differential scanning calorimetry (DSC) was performed at different pH values. Figure 4a shows the pH dependence of the thermal unfolding transitions for Cip1, with an optimum thermal stability at approximately pH 4. As can be seen in the figure, the reversibility of the thermal unfolding transitions is also dependent upon pH with a percentage reversibility that is at its greatest between pH 7.3 and 8.6. Figure 4b shows the temperature dependence and reversibility of the thermal unfolding of Cip 1 in the absence and presence of EDTA. The study was performed at pH 6.8 since the structure of Cip 1 was obtained from crystals grown at pH 7.0, and pH 6.8 was closest to the crystallisation pH of all the buffers used. The thermal melting point of Cip1 at pH 6.8 was 66.1±0.3°C and 67.3±0.9°C in the absence and presence of 5 mM EDTA, respectively. The effect of EDTA on the thermal melting midpoint (Tm) is therefore negligible. However, a larger effect of EDTA addition was seen in the reversibility of the unfolding transition; the percentage reversibility was decreased from 58.9±1.1% to 30.7±3.1% when Cip1 is thermally unfolded in the presence of 5 mM EDTA. Thus, it is clear that the removal of the calcium ion by addition of EDTA significantly affects the reversibility of the unfolding transition and this is consistent with a structural role for calcium in Cip1.

**Figure 5:**
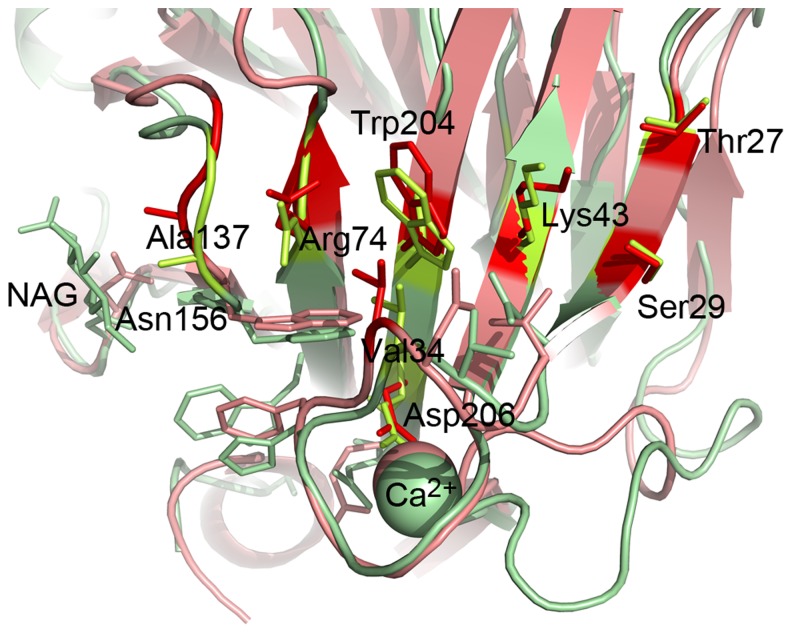
The “grip” motif in Cip1 compared to glucuronan lyase from *H.* *jecorina*. The grip motif is a conserved region in Cip1, both sequentially and structurally, here showing Cip1 (green) superposed to the glucuronan lyase from *H. jecorina* (red). In these two structures, there is a string of homologous residues that are located across the “palm” β-sheet (bright colours). The loop representing the “bent fingers” participates in binding a calcium ion represented as a sphere. The conserved coordinating aspartate is also shown in bright colours. Asn156 in Cip1 binds a N-acetyl glucosamine molecule but the equivalent residue in the glucuronan lyase is a non-glycosylated aspartate. Many of the residues that are not identical are yet similar in physical properties.

**Figure 6:**
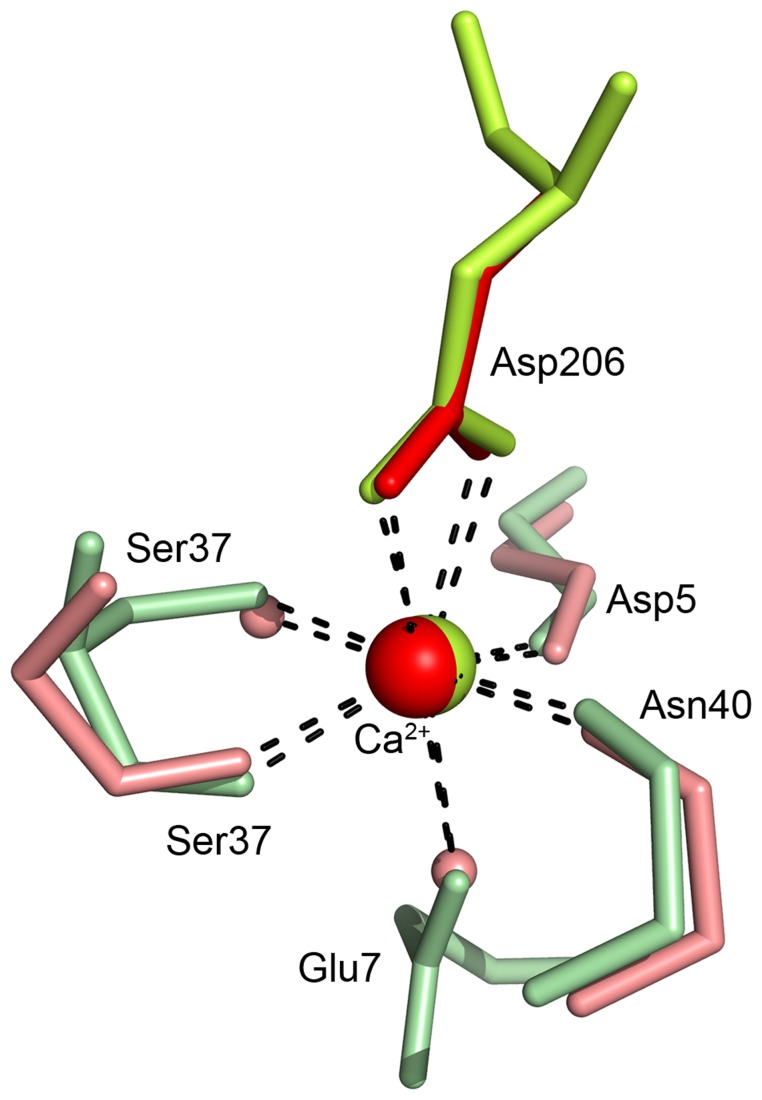
The calcium binding site in Cip1 compared to glucuronan lyase from *H.* *jecorina*. The calcium binding site found in the Cip1 structure. Cip1 structure (green) superposed to the glucuronan lyase structure from *H. jecorina* (red). Asp206 is shown in bright colours since it is sequentially and structurally conserved and it coordinates the calcium ion with the two side chain oxygen atoms (also see Figure 8). All coordination distances are between 2.3 Å and 2.6 Å.

As can be seen in Figures 2 and 5, the calcium ion is located in a pocket between C-terminal β-strand fifteen (Asn201-Ala211), the N-terminal loop (Phe6-Trp15) that connects β-strands 1 (Ile2-Asp4) and 2 (Pro16-Ser18) and the “bent fingers” loop (Thr32-Ser41) that connects β-strands 3 (Thr27-Asp31) and 4 (Met42-Gly46). Calcium ions have characteristic coordination spheres of six or seven ligands, which are most often the carboxylic or the carboxamide of aspartic or glutamic acid. The calcium ion in the structure of Cip1 is hepta-coordinated and bound to seven oxygen ligands (Figure 6). The side chains of Glu7, Ser37 and Asp206 provide four of these, the latter bindjng in a bidentate mode with both oxygen atoms. The other three ligands consist of the carboxylic main chain oxygen atoms of Asp5, Ser37 and Asn40.

## Discussion

### Lyase activity measurements

The two closest structural homologs of Cip1, CsGL, a glucuronan lyase from *H. jecorina* and vAL-1, an alginate lyase from the *Chlorella* virus, are both classified lyases. As previously mentioned, lyase activity was tested for Cip1 with the substrate glucuronan. Disappointingly, the apparent lyase activity detected was too low to be considered convincing. However, it is possible that the experiment was not performed at an optimal pH for the enzymatic reaction, or that the utilised substrate had a low binding affinity for the enzyme, thus making it energetically unfavourable to fit into a plausible active site. We should note that Cip1 was characterised with the same substrate and at the same pH optimum as the known *H. jecorina* glucoronan lyase. Determination of Cip1 lyase activity might be a matter of finding the correct substrate and/or adjusting the pH.

### Features and comparative analysis of Cip1 to other protein structures

A structure similarity search with the structure coordinates of Cip1 against all known and public protein structures revealed a high degree of structural similarity between Cip1 and the protein structures of CsGL, a glucuronan lyase from *H. jecorina* (PDB ID: 2ZZJ), [12] and vAL-1, an alginate lyase from the *Chlorella* virus (PDB ID: 3A0N) [13]. The root-mean-square deviation (RMSD) values for these structures when superposed with the Cip1 structure, utilising the program Lsqman [14], were 1.54 Å (for 158 matched Cα atoms) and 1.98 Å (for 143 matched Cα atoms), respectively. Some similarity was also found with the structure of CsCBM27-1, a protein with a CBM of family 27 from *Caldicellulosiruptor saccharolyticus* (PDB ID: 1PMH) in complex with a mannohexaose molecule [10]. Two regions stand out when comparing Cip1 to these three structures, namely the two regions described above as the “grip” motif and the plausible active site cleft.

**Figure 7:**
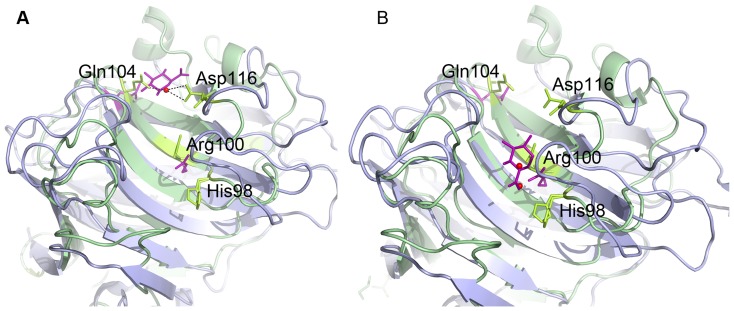
Comparison of Cip1 to alginate lyase from *Chlorella* virus at pH 7 and pH 10. Superposition of Cip1 from *H. jecorina* (green) to the alginate lyase from *Chlorella* virus (blue) and the interactions with bound D-glucuronic acid (violet) at A) pH 7 and B) pH 10. The residues are numbered according to the Cip1 structure. Plausible catalytic residues are brightly coloured in the figure. Water molecules are depicted in red and belong to the structure of Cip1. Panel **A** displays the alginate lyase structure at pH 7, the D-glucuronic acid interacts with the glutamine at the top of the active cleft. The corresponding glutamine in Cip1 (Gln104) instead forms a hydrogen bond to a water molecule, which is also bound by Asp116, a residue that has dual conformations in Cip1. Panel **B** displays the alginate lyase structure at pH 10, the D-glucuronic acid interacts with Arg100 at the lower end of the cleft. Both Asp116 and His98 in Cip1 show dual conformations pointing toward this position which may be an indication that the region is dynamic and that these residues are somehow involved in substrate binding. Asp116 and His98 do not have any equivalents in the lyase structure.

**Figure 8:**
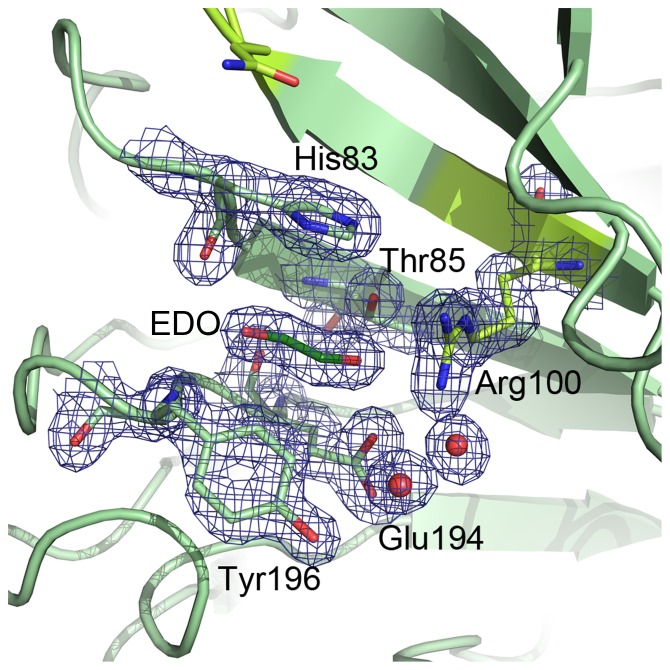
Cip1 pocket that binds ethylene glycol. With Arg100 (lime green) forming one of the walls, Thr85, Glu194, His83 and Tyr196 together create the rest of a small pocket on one side of the plausible active site cleft, in which an ethylene glycol (dark green) is found in the structure of Cip1. To facilitate comparison of figures, Gln104 is also shown (lime green). Electron density is contoured at a level of 1.0 sigma (0.4 electrons/Å^3^).

Cip1 has two potential substrate binding residues in common with the *Chlorella* alginate lyase in the potential substrate-binding cleft. One is Gln104, corresponding to Gln120 in the alginate lyase. This residue interacts with bound D-glucuronic acid in the structure of the *Chlorella* alginate lyase at pH 7 (PDB ID: 3A0N) (Figure 7a). The *H. jecorina* glucuronan lyase also has a glutamine at this position but no substrate was modelled into the structure. The other potential substrate-binding residue is an arginine at position 100 in Cip1, corresponding to Arg116 in the alginate lyase. This residue is located at the bottom of the active site cleft in the *Chlorella* alginate lyase and interacts with the bound substrate at pH 10 (PDBID: 3IM0) (Figure 7). Instead of an arginine, the *H. jecorina* glucuronan lyase has a methionine at this position. Two Cip1 residues, Asp116 and His98, are located in the vicinity of the active site glutamine and arginine and both are modelled with dual conformations, which indicate that the region is dynamic (Figure 7). Gln104, Arg100, His98 and Asp116 are marked in orange in the sequence alignment in Figure 1. While the two lyase structures described above show many charged residues lining the potential active site cleft, with the most hydrophobic ones being tyrosines, CsCBM27-1 is dependent upon three tryptophan residues to bind its mannohexaose substrate [10]. Since the residues lining the plausible active site cleft in Cip1 are mostly charged and correlate well with the lyases it is, as discussed above, possible that Cip1 may have lyase activity. This could offer an explanation as to why the many different binding and glycoside hydrolase activity studies performed for Cip1 were not successful. One possible interaction site is a region where an ethylene glycol molecule is found bound in the Cip1 structure (Figure 8). Apart from the previously mentioned Arg100 in Cip1, the ethylene glycol molecule interacts with Thr85 and Glu194 (hydrogen bonds), as well as both main chain (hydrogen bonds) and side chain (stacking and packing) interactions with His83 and Tyr196 (Figure 8). Interestingly, all of these residues are completely conserved in all Cip1 homologs, in fungi as well as bacteria, except for Thr85 that can also be a serine or an alanine (Figure 1). However, when structurally comparing this region in Cip1 to the glucuronan and alginate lyase structures, very little structural similarity is found. It is thus possible that these conserved ethylene glycol-interacting residues are somehow involved in the specific Cip1 activity, perhaps when interacting with a substrate molecule.

The “grip” motif is very similar when comparing Cip1 to the *H. jecorina* glucuronan lyase (PDB ID 2ZZJ), having many residues in common, as well as a bound calcium ion (Figure 5). The calcium-binding site is described in further detail below. As can be seen in Figure 5, the homologous residues are located in a string across the β-sheet palm, and many neighbouring residues that are not identical are still similar in type and structure. The identical and similar residues in the “grip” region are coloured in green in the sequence alignment (Figure 1). The alginate lyase does not show the same degree of similarity to Cip1 in this region and it does not bind calcium. Cip1 was treated with EndoH prior to crystallisation, trimming the glycosylation to leave only one bound N-acetyl glucosamine molecule. This can be seen in the structure, where Asn156 binds a NAG on the surface of Cip1 just outside the “grip” region (Figure 5). The *Chlorella* alginate lyase also has an asparagine at this position whereas the *H. jecorina* glucuronan lyase has an aspartate.

To summarise, Cip1 has two major regions with structural similarity to lyases; the potential active site cleft, which resembles that of an alginate lyase from the *Chlorella* virus, and the “grip” motif, which binds calcium and resembles that of a glucuronan lyase from *H. jecorina.* Based on these facts it can be hypothesised that Cip1 is a lyase, although no significant lyase activity was measured in this study.

### The calcium binding site

Inspection of the structural similarity search top hit, the *H. jecorina* glucuronan lyase structure (PDB ID2ZZJ), did show that this structure has a calcium ion bound in an equivalent position to the one found in the Cip1 structure. Superposition of the Cip1 and the *H. jecorina* glucuronan lyase structure (2ZZJ) shows that these structures are almost identical in that region, differing only in that two side chain ligands in Cip1 (Glu7 and Ser37) are exchanged for water molecules in glucuronan lyase structure (2ZZJ). Sequence alignment shows that the coordinating residues Asp206 and Asp5 (Asp7 and Asp222 in 2ZZJ, respectively) are conserved. Figure 6 shows the calcium ion with coordinating residues, the structure of Cip1 superposed to that of the glucuronan lyase from *H. jecorina*. Figure 1 shows a sequence alignment of all currently known Cip1 homologs and the residues coordinating the calcium ion are marked in yellow.

The calcium ion is situated at a critical position in the Cip1 structure; the loops that interact with it are located close to the N-terminus on the convex side of the molecule, exposed to the bulk solvent. Since calcium generally has a larger flexibility in accepting more variable and irregular coordination geometries than similar ions [15], calcium can make multiple interactions with these loops, thereby stabilising the structure in that region. In addition to the interaction with the N-terminus, the calcium ion has indirect interaction with the C-terminus via Asp206 (Figure 6).

### Concluding remarks

The presence of various Cip1 homologs in diverse microorganisms and the co-regulation of Cip1 expression with the major cellulases in *H. jecorina* indicate that the protein Cip1, with yet unknown function, plays an important role in degradation of and/or the binding to cellulosic substrates. However, the current biochemical study did not reveal any significant activity or binding on the carbohydrates that were tested, beyond the previously reported binding of cellulose and xyloglucan by CBMs in family 1 [7]. Still, the modular structure and the expression data point towards a function in biomass degradation. A structural similarity search using the crystal structure of Cip1 generated two hits with high scores and published structures, a glucuronan lyase from *H. jecorina* (PDB ID: 2ZZJ) and an alginate lyase from the *Chlorella* virus (PDBID: 3GNE). Parts of these structures show strong resemblance to Cip1, indicating that Cip1 may have lyase activity. Although no significant lyase activity was found with the tested carbohydrate source, we are now a few steps closer to knowing the true role of Cip1 in the biomass degradation performed by *H. jecorina.* The Cip1 structure may be used in the future as a basis for further biochemical characterisation of Cip1 and homologous enzymes.

## Materials and Methods

### Subtract hybridisation of lactose induced *H. jecorina* strain QM6a

RNA isolation and *Escherichia coli* cDNA library preparation of lactose-induced *H. jecorina* strain QM6a fermentation was performed as described by Foreman *et al.* [6] *E. coli* transformants with *H. jecorina* cDNA clones were grown over night at 37°C in TY (Trypton Yeast) medium (10 g/L yeast (Bacto); 16 g/L trypton (Bacto); 5 g/l NaCl (Fluka) pH7), including 100 µg/ml ampicillin, in 384 well microtitre plates. The microtitre plates were replicated onto 20×20 cm Hybond+ filters (Amersham Pharmacia Biotech, Amersham, United Kingdom), placed on large agar petri-dish plates including TY agar-medium (1.5% agar) and 100 µg/ml ampicillin, and grown over night at 37°C. *E-coli* colonies growing on the hybridisation filters were lysed and fixed by putting the membrane onto 0.5 M NaOH solution and washed five times with a saline-sodium citrate (SSC) solution, and then used for hybridisation. Hybridisation was performed using an ECL system from Amersham Pharmacia Biotech, Amersham, United Kingdom (RPN3000), according to the described standard protocol (“Direct nucleic acid labelling and detection”).

PCR fragments of carbohydrate binding module (CBM) containing proteins were prepared from genomic *H. jecorina* QM6a preparations. Degenerated PCR primers (Table S1, supplementary material) were used to obtain PCR fragment of known *H. jecorina* CBMs using a touchdown PCR reaction performed according to the following PCR protocol: 10 cycles of; 1 minute at 94°C; 1 minute and 30 seconds at 65°C (ramping to 50°C during the next 9 cycles); and 1 minute at 72°C; followed by 25 cycles of; 1 minute at 94°C; 1 minute and 30 seconds at 50°C; and 1 minute at 72°C. The PCR mixture was prepared in a volume of 50 µl containing: template *H. jecorina* QM6a: 100 ng; Primers: 10 µM 1 µL FRG164; 100 µM 1 µL/FRG165, FRG166 or FRG167; 2.5 units platinum TAQ polymerase; 5 µL 10× TAQ buffer; 1.5 µL MgCl_2_; 1 µL 10 mM dNTP's.

Nine PCR fragments of genes coding for the catalytic domain of *H. jecorina* proteins known to contain a CBM were prepared using a standard PCR protocol (primers used are listed in Table S1, supplementary material). All nine PCR fragments were mixed equally and labelled using the ECL system as described by Amersham, and used as probes for hybridisation experiments. Hybridisation experiments were performed as described in the ECL manual protocol.

### Cloning and expression of Cip1

The obtained *cip1* cDNA sequence was cloned into the gene expression plasmid pTREX3g, according to the method described in US patent US2007/0128690. The Cip1 protein was expressed in a “deleted” version of the *H. jecorina* strain QM6a in which the four major cellulase genes (*cbh1/cel7a*, *cbh2/cel6a*, *egl1/cel7b*, and *egl2/cel5a*) have been disrupted, as described [16]. The “deleted” QM6a strain was transformed with a circular plasmid carrying the *cip1* gene behind the strong *H. jecorina cel7a* promoter. The resultant *H. jecorina* strain was grown at 25°C in a batch-fed process with lactose (1.6 g/L) as carbon source and inducer using a minimal fermentation medium essentially as described [17]. Initially, 0.8 L of culture medium containing 5% glucose was inoculated with 1.5 ml of *H. jecorina* spore suspension. After 48 hours, the culture was transferred to 6.2 L of the same media in a 14 L fermentor (*Biolafitte, Princeton, NJ*). One hour after the glucose was exhausted, a 25% (w/w) lactose feed was started in a carbon-limiting fashion so as to prevent its accumulation. The pH during fermentation was maintained in the range of 4.5–5.5. After 165 hours of growth 17 g/L total protein was expressed, and Cip1 constituted more than 80% of the total secreted protein, as judged by SDS-PAGE (not shown). The expression host *H. jecorina* was removed from the culture media by filtration.

### Protein purification

A cell free supernatant sample of Cip1 was purified by hydrophobic interaction chromatography on a BioCAD Sprint Workstation (Perspective Biosystems, Cambridge, MA) by the following protocol: A hydrophobic interaction chromatography column, Poros 20 HP2 10 column (Perspective Biosystems, Cambridge, MA), was equilibrated with 5 column volumes (CV) of 0.5 M (NH_4_)_2_SO_4_/0.02 M NaH_2_PO_4_, pH 6.80; 30 ml of the concentrated Cip1 protein sample, with an addition of 0.5 M (NH_4_)_2_SO_4_, was applied to the column; the column was washed with 10 CV of 0.5 M (NH_4_)_2_SO_4_/0.02 M NaH_2_PO_4_, pH 6.80; followed by a protein elution step using a 5 CV gradient from the initial loading buffer to 0.02 M NaH_2_PO_4_, pH 6.80. The most pure Cip1-containing fractions after the hydrophobic interaction chromatography purifications, as judged by SDS-PAGE, were pooled and concentrated to a final volume of 13 mL, using Millipore centrifugal concentration units, with a 5 kDa membrane molecular weight cut-off (Biomax 5K; Millipore, Bedford, MA). The concentrated Cip1 sample was applied to a Superdex75 Hi-Load 26/60 size exclusion column, (GE Healthcare), using a running buffer of 0.02 M NaH_2_PO_4_, pH 6.80. The eluted fractions were analysed by SDS-PAGE (data not shown) and the purity of the Cip1 protein was estimated to be greater than 95% at this point.

For the purpose of crystallisation experiments, deglycosylated Cip1 core domain was prepared from the purified intact protein using the deglycosylation procedure described previously for *H. jecorina* Cel7A [18]. A solution of 20 mg Cip1 in 10 ml of 100 mM NaAc/5 mM Zn(Ac)_2_ at pH 5.0, was incubated for 48 hours at 37°C with jack bean α-mannosidase (Sigma-Aldrich) and *Streptomyces plicatus* endoglycosidase H (EndoH, kind gift from DuPont IB, Palo Alto) at a final ratio of Cip1/mannosidase or Cip1/EndoH of 1/80 and 1/40 (w/w), respectively. Next, Cip1 core domain was prepared by partial proteolytic cleavage of the protein using the protease papain (Sigma Aldrich) at a final Cip1/papain ratio of 1/100 (w/w), and 48 hours incubation at room temperature. The deglycosylated and proteolytically produced Cip1 core domain protein was purified by anion exchange chromatography on a Source 30Q column (GE Healthcare) at pH 5.0 using a 10 mM to 100 mM NaAc gradient. The eluted fractions corresponding to Cip1 core domain protein were collected and loaded onto a Superdex-200 Hiload 16/60 size exclusion column (GE Healthcare), using a running buffer consisting of 10 mM NaAc pH 5.0. The fractions containing the Cip1 core domain protein were pooled, and the purity of the protein sample was estimated to be greater than 95%, as judged by SDS-PAGE (not shown). The purified Cip1 core domain protein sample was dialysed and concentrated to a final protein concentration of 20 mg/ml in 20 mM HEPES buffer, pH 7.0, using a Vivaspin concentrator (Sartorius Stedim Biotech) with a polyethersulphone membrane with a 5 kDa membrane molecular weight cut-off.

For the biochemical characterisation two additional purification steps were introduced: one additional anion exchange chromotography step using a Source 30Q column as described above, and a subsequent affinity purification using 4-aminobenzyl β-D-glucoside bound to Sepharose 4B (GE Healthcare), according to the protocol described in [19], to remove potential residual β-glucosidase activity. This purification was performed for both intact Cip1 and Cip1 core domain. The affinity column was equilibrated with 100 mM NaAc, pH 5.0 containing 200 mM NaCl. After applying the partially purified Cip1, the column was washed with the equilibration buffer and bound protein was eluted with an elution buffer containing 100 mM glucose and 200 mM NaCl in 100 mM NaAc, pH 5.0. The Cip1 protein was found in the flow-through fraction and did not show any potential β-glucosidase or endoglucanase residual activity on the chromogenic substrates 2-chloro-4-nitrophenyl-β-D-glucoside and -β-cellobioside. The concentration of the purified protein was determined with the Bradford assay [20] using bovine serum albumin as standard.

### Biochemical characterisation of Cip1

Lichenan (from *Cetraria islandica*), laminarin (from *Laminaria digitata*), birchwood xylan, barley glucan and polygalacturonic acid were obtained from Sigma-Aldrich, tamarind xyloglucan, wheat flour arabinoxylan and locust bean galactomannan from Megazyme, carboxymethylcellulose from BDH Chemicals, cotton linters and apple pectin from Fluka, Avicel cellulose from Macherey-Nagel and cello-oligosaccharides from Merck. Phosphoric acid swollen cellulose was prepared as described in [21], and the 2-chloro-4-nitrophenyl-β-glycosides (CNPG, CNPG_2_ and CNPLac), were synthesised as described in [22], [23].

All activity and binding assays were performed at 37°C in 100 mM NaAc buffer, pH 5.0, except for the hydrolysis experiments with CNP-β-glycosides, which were performed in 100 mM sodium phosphate buffer, pH 5.7. The release of 2-chloro-4-nitrophenol was monitored continuously by measuring the absorbance at 405 nm. The hydrolysis of 0.5 mM cellopentaose with 0.7 µg Cip1 was followed by High Performance Anion Exchange Chromatography with Pulsed Amperometric Detection (HPAEC-PAD) on a Dionex ICS3000 system (Dionex), according to the manufacture's procedures. Gel diffusion assays with 0.05% (w/v) carboxymethylcellulose, birchwood xylan, arabinoxylan, galactomannan, laminarin or lichenan added to 0.5% (w/v) agarose, and gel electrophoresis with native polyacrylamide gels incorporating 0.25% (w/v) carboxymethylcellulose, xyloglucan, lichenan, laminarin, birchwood xylan, galactomannan, arabinoxylan, barley glucan or 0.01% apple pectin, or polygalacturonic acid, were performed using methods identical to those described in [24], [25]. In the latter assay *H. jecorina* cellobiohydrolase Cel7A (both intact and core domain enzyme without the carbohydrate binding module) and bovine serum albumin were added as control proteins. Adsorption experiments (pH 5.0, 20°C) of intact Cip1 and proteolytic core domain Cip1 onto Avicel cellulose suspensions were performed as described in [26] by measuring the absorbance at 280 nm.

Cellulase activity on cotton linters and phosphoric acid swollen cellulose were assayed at 37°C in 1.2 ml reaction mixtures (2% substrate in 40 mM NaAc buffer, pH 5.0). The assays were performed with 0.1 µM *H. jecorina* Cel7A, 0.1 µM Cip1, and a mixture of both enzymes. Samples were taken after 5 minutes and 17 hours. An excess of *Aspergillus niger* cellobiase (Sigma-Aldrich) was added to 200 µl sample, and the total glucose concentration was measured with the coupled glucose oxidase (from *Aspergillus niger*; Sigma-Aldrich)-peroxidase (from Horse radish; Roche) assay using 2,2′-azino-di(3-ethylbenzthiazoline-6-sulphonate (ABTS, Roche) as chromogen [27]. Activities were expressed in µM glucose formed.

Measurements to test lyase activity for Cip1 were performed as described previously by Konno *et al.* [28]: *i.e.* at 50°C, in sodium phosphate buffer (50 mM) using glucuronan (0.5% w/v) as a substrate (kind gift from Dr. Kiyohito Igarashi, Tokyo University, Japan) and at the pH optimum (6.5) for the *H. jecorina* glucuronan lyase.

### Crystallisation and Data Collection

To determine the homogeneity and the oligomerisation state of the Cip1 protein, dynamic light scattering experiments were carried out using a DynaPro 801 TC instrument (Wyatt Technology corp., Santa Barbara, USA). The impact of temperature on the homogeneity of Cip1 was determined by taking DLS spectra at regular temperatures intervals, ranging from 5 to 45°C, using 100 uL samples of Cip1, 5 mg/mL in 20 mM HEPES buffer pH 7.0. Initial DLS spectras were taken at 5°C and the temperature was then increased with 5 degrees increment before a new spectrum was recorded. The protein sample was allowed to equilibrate for 20 minutes at each new temperature before a new DLS spectrum was recorded at this temperature.

Cip1 crystals were grown using the hanging-drop vapour diffusion method [29] at 4°C. Crystallisation drops were prepared by mixing equal amount of protein solution, containing 20 mg/mL of protein, and crystallisation solution, containing 20 mM HEPES pH 7.0, and 1–1.5 M ammonium sulphate. Crystals grew within one week after preparation of the crystallisation drops. Prior to x-ray data collection, crystals were flash frozen in liquid nitrogen using the crystallisation solution with 30% PEG 3350 added as a cryo-protectant. Initially, Cip1 crystals were soaked into a lead-containing solution to use the data collected from these crystals for phasing by Multi-wavelength Anomalous Dispersion (MAD) or Single-wavelength Anomalous Dispersion (SAD), as appropriate. The crystals gave strong x-ray diffraction, but no anomalous signal from lead was obtained from this data. However, the high quality of the crystal led us to make an attempt to solve the structure by sulphur-SAD, and so a data set was collected to a resolution of 2.0 Å, at λ = 1.771 Å. X-ray diffraction data collection was performed on the bending magnet beam line BM-14 at the European Synchrotron Radiation Facility (ESRF), Grenoble, France. Since the Cip1 crystals did not apparently seem affected by radiation, a great number of diffraction images could be collected to obtain better redundancy of the data, enabling phasing by sulphur-SAD. A total of 720 consecutive diffraction images (720° of data) were collected from one Cip1 crystal, which resulted in an average data multiplicity greater than 18 and completeness of 100%.

### Structure determination and model refinement

The sulphur-SAD data set was submitted to SHELXD [30], [31] and the program successfully found the position of 13 sites. The position of these 13 sites were further refined, and the initial phases were calculated, using the program SHARP [32]. After the refinement of the 13 sites in SHARP the quality of the electron density maps were excellent. The overall phasing power was 1.36, yielding an overall figure of merit 0.41 and 0.12 for acentric and centric reflections, respectively. The phases obtained from SHARP were further improved by solvent flattening using the program SOLOMON [33]. Using the obtained improved phases, the automated protein building and refinement program ARP/wARP, [34] could automatically build the complete structure, *i.e*. 218 residues. The resolution of this Cip1 sulphur-SAD data was only 2.0 Å and therefore two additional native data sets (high and low resolution from another crystal) were collected. These additional Cip1 native data sets were merged, and the resolution of the Cip1 structure could be extended to the resolution limit of these, 1.5 Å, by refining the initially built 2.0 Å structure against the merged native dataset using rigid body refinement. Details of crystallographic data collection and phasing statistics are summarised in Table 1.

The datasets were processed using DENZO and SCALEPACK. [35] Details of diffraction data collection and processing statistics are presented in Table 1. The Cip1 crystals belong to the space group P2_1_2_1_2_1_ with unit-cell parameters of a = 55.4 Å, b = 57.5 Å and c = 74.6 Å, giving a calculated V_m_ of 2.5 [36] with an estimate of one molecule in the asymmetric unit. Refinement was performed using REFMAC5 [37] in the CCP4 package [38]. For cross-validation purposes a set of 5% of the x-ray data was excluded from the refinement for R_free_ [39] calculations. Solvent molecules were added automatically to the structure model using ARP/wARP [34] and by manual modelling. Throughout the refinement, 2mF*_o_*-DF*_c_* and mF*_o_*-DF*_c_* σ_A_-weighted maps [40] were inspected and the structure models manually adjusted in O [41] and Coot [42]. Structure model and refinement statistics are presented in Table 1. The RMSD values between Cip1 and structures found by homology searches were calculated utilising Lsqman [14] with a value of 3.5 Å for C_α_ cut-offs. Structure coordinates and structure factors for the final Cip1 structure model have been deposited to the Protein Data Bank [43] (accession number 3ZYP).

### Elemental analysis of Cip1 by micro-PIXE

The metals bound to Cip1 were identified by particle-induced X-ray emission spectrum (PIXES) using the ion beam analysis laboratory at the university of Surrey, Guilford, UK [44], [45]. The protein sample was prepared to a final concentration of 3 mg/ml in 10 mM sodium acetate buffer, pH 5.0, and the metals bound to Cip 1 were identified using either beam energy of 1.5 MeV or 2.5 MeV. The beam energies of 1.5 MeV and 2.5 MeV were selected for sensitivity towards magnesium and other elements above iron, respectively. The PIXE spectrum for Cip1 and the metal ions present were identified by comparison with the minimum detectable limit (MDL) of the smallest measurable atomic ratio for that element.

### Differential Scanning Calorimetry

Excess heat capacity curves of Cip1 were measured using an ultra sensitive scanning high-throughput micro-calorimeter, VP-Cap DSC (MicroCal, Inc., Northampton, MA). Samples of Cip 1, 0.5 mg/mL, were scanned from 35°C to 90°C over a pH range from 3.9 to 8.7 in the absence and presence of 5 mM EDTA, using standard buffers purchased from Hampton Research, Inc. Ca. The dependence of the thermal melting points for Cip1 on the scan rate was assessed over a scan rate of 90 to 200°C/hr. The thermal melting point for Cip 1 was dependent on the scan rate, and the scan 200°C/hr was used to minimise any artefacts that may result from aggregation. The reversibility of the thermal unfolding process was assessed by rescanning the same sample after cooling. The thermal melting midpoint (Tm) of the DSC curves was used as an indicator of the thermal stability, and was obtained using the software Origin 7.0 (Origin Lab, MA). Under the conditions where the thermal unfolding process was reversible, the percentage reversibility was calculated by comparing the ratio of the amplitude of the forward scan by the amplitude of the rescan. The amplitudes for the heat capacity curves were obtained by fitting the data using the software Peakfit v. 4.12 (Seasolve Software, Inc, MA).

## Supporting Information

Figure S1 **Pairwise identity percentages of all currently known Cip1 homologs.** The figure shows pairwise identity percentages of all currently known Cip1 homologs. The grey area shows the fungal identity couples. The sequences (EMBL Genbank access numbers indicated in parentheses) are: seq. 1, *Hypocrea jecorina* Cip1 (AAP57751); seq. 2, *Pyrenophora teres f teres* 0–1 (EFQ89497); seq. 3, *Pyrenophora tritici repentis* (XP_001937765); seq. 4, *Chaetomium globosum* (XP_001228455); seq. 5, *Chaetomium globosum* (XP_001222955); seq. 6, *Phaeosphaeria nodorum* SN15 (XP_001790983); seq. 7, *Podospora anserina* S mat+ (XP_001906367); seq. 8, *Magnaporthe oryzae* 70-15 (XP_365869); seq. 9, *Nectria haematococca* mpIV (XP_003039679); seq. 10, *Gibberella zeae* PH-1 (XP_386642); seq. 11, *Haliangium ochraceum* DSM 14365 (YP_003266142); seq. 12, *Herpetosiphon aurantiacus* ATCC 23779 (YP_001545140); seq. 13, *Catenulispora acidiphila* DSM 44928 (YP_003114993); seq. 14, *Streptomyces coelicolor* A3(2) (NP_629910); seq. 15, *Streptomyces lividans* TK24 (ZP_05523220); seq. 16, *Streptomyces sp.* ACTE (ZP_06272077); seq. 17, *Streptomyces sviceus* ATCC 29083 (ZP_06915571); seq. 18, *Streptomyces sp.* e14 (ZP_06711846); seq.19, *Actinosynnemma mirum* DSM 43827 (YP_003101274); seq. 20, *Amycolatopsis mediterranei* U32 (YP_003767350); seq. 21, *Streptomyces violaceusniger* Tu 4113 (ZP_07602526); seq. 22, *Cellulomonas flavigena* DSM 20109 (YP_003638201); seq. 23, *Micromonospora aurantiaca* ATCC 27029 (YP_003835070); seq. 24, *Micromonospora sp.* L5 (YP_004081730).
(TIF)

Table S1 **Gene-specific (catalytic domain) and degenerate (CBM) primers of the known CBD containing genes in** ***H. jecorina*** **(Genomic DNA of strain QM6A).**
(PDF)
