# Supplementary figures and images for: The Crystal Structure of the Core Domain of a Cellulose Induced Protein (Cip1) from Hypocrea jecorina, at 1.5 Å Resolution
Source: PLoS One. 2013 Sep 5;8(9):e70562. doi: 10.1371/journal.pone.0070562 (PMC3764139; doi:10.1371/journal.pone.0070562)

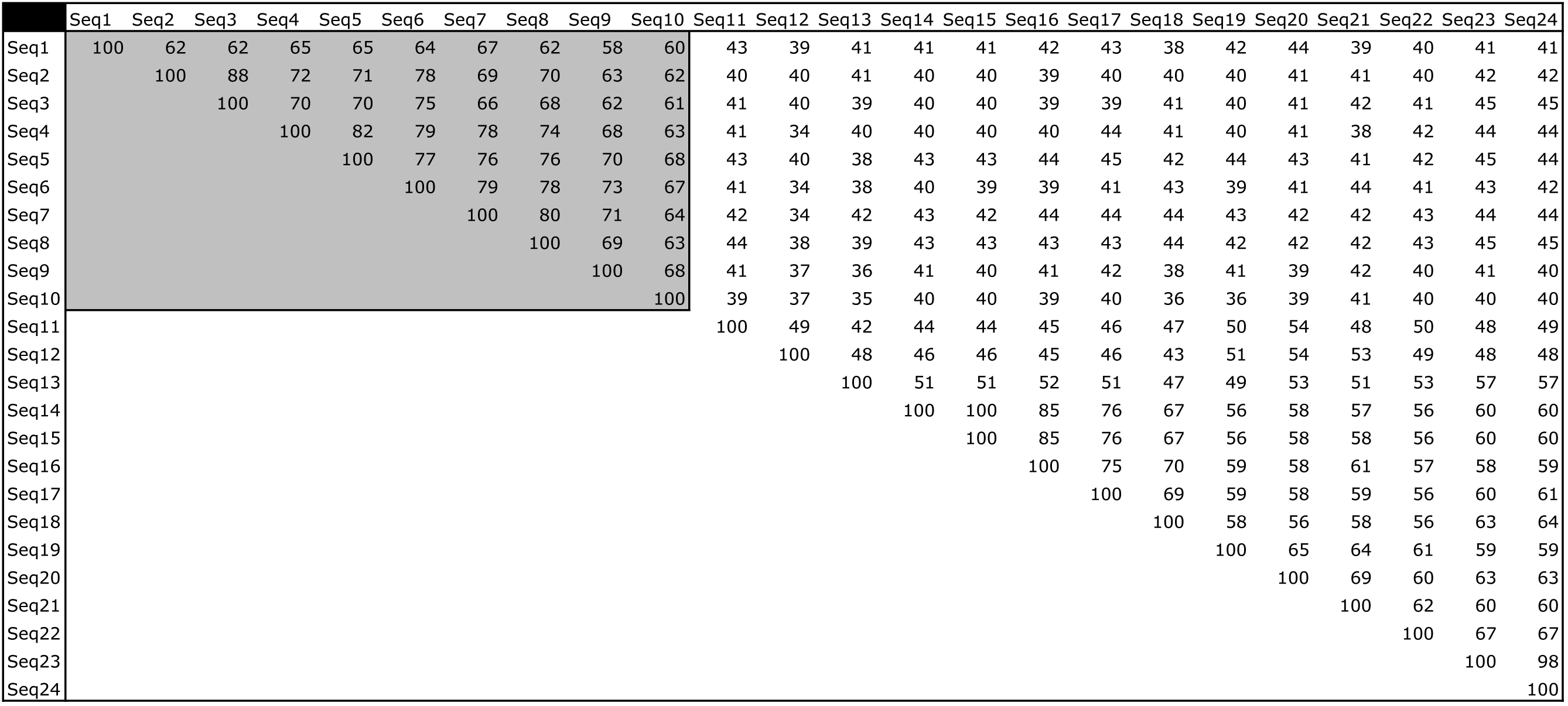

Supplement: Figure S1 — Pairwise identity percentages of all currently known Cip1 homologs. The figure shows pairwise identity percentages of all currently known Cip1 homologs. The grey area shows the fungal identity couples. The sequences (EMBL Genbank access numbers indicated in parentheses) are: seq. 1, Hypocrea jecorina Cip1 (AAP57751); seq. 2, Pyrenophora teres f teres 0–1 (EFQ89497); seq. 3, Pyrenophora tritici repentis (XP_001937765); seq. 4, Chaetomium globosum (XP_001228455); seq. 5, Chaetomium globosum (XP_001222955); seq. 6, Phaeosphaeria nodorum SN15 (XP_001790983); seq. 7, Podospora anserina S mat+ (XP_001906367); seq. 8, Magnaporthe oryzae 70-15 (XP_365869); seq. 9, Nectria haematococca mpIV (XP_003039679); seq. 10, Gibberella zeae PH-1 (XP_386642); seq. 11, Haliangium ochraceum DSM 14365 (YP_003266142); seq. 12, Herpetosiphon aurantiacus ATCC 23779 (YP_001545140); seq. 13, Catenulispora acidiphila DSM 44928 (YP_003114993); seq. 14, Streptomyces coelicolor A3(2) (NP_629910); seq. 15, Streptomyces lividans TK24 (ZP_05523220); seq. 16, Streptomyces sp. ACTE (ZP_06272077); seq. 17, Streptomyces sviceus ATCC 29083 (ZP_06915571); seq. 18, Streptomyces sp. e14 (ZP_06711846); seq.19, Actinosynnemma mirum DSM 43827 (YP_003101274); seq. 20, Amycolatopsis mediterranei U32 (YP_003767350); seq. 21, Streptomyces violaceusniger Tu 4113 (ZP_07602526); seq. 22, Cellulomonas flavigena DSM 20109 (YP_003638201); seq. 23, Micromonospora aurantiaca ATCC 27029 (YP_003835070); seq. 24, Micromonospora sp. L5 (YP_004081730). (TIF) [file pone.0070562.s001.tif]
