# Supplementary material for: The Crystal Structure of the Core Domain of a Cellulose Induced Protein (Cip1) from Hypocrea jecorina, at 1.5 Å Resolution
Source: PLoS One. 2013 Sep 5;8(9):e70562. doi: 10.1371/journal.pone.0070562 (PMC3764139; doi:10.1371/journal.pone.0070562)
Supplement: Table S1 — Gene-specific (catalytic domain) and degenerate (CBM) primers of the known CBD containing genes in H. jecorina (Genomic DNA of strain QM6A). (PDF) [file pone.0070562.s002.pdf]

gene (catalytic domain) specific primers				
**Gene**	**orientation**	**primer**	**sequence**	
cbh1	forward	FRG168	CTC CTC CAC ACC CGG TGC CG	
	reverse	FRG169	TGC TGC CAA TGG GTC CG	
cbh2	forward	FRG170	ACG TAT TCA GGC AAC CC	
	reverse	FRG171	GCA GTG GCC ATG GCT CC	
eg1	forward	FRG172	CCA GTA CAT GAA CTG GC	
	reverse	FRG173	AGA CCC AAT GTC TCC CC	
eg2	forward	FRG184	CGA ATT GTG CTC CTG GC	
	reverse	FRG185	GTG GTT GGA CCG GAT GG	
eg4	forward	FRG176	CCT ACC GTG GTA TCA GG	
	reverse	FRG177	TGG TTC TGC TGG TCG GG	
eg5	forward	FRG178	CAT TTC GAC ATC ATG GC	
	reverse	FRG179	CTG TCC CAC GCA GAG GC	
axe1	forward	FRG180	CCG GCT GGC TTC GTC TG	
	reverse	FRG181	TGG CCG TAA CCT TGG TG	
ß-mannase	forward	FRG182	CCT CTC TCA CGA CTC GC	
	reverse	FRG183	GTT CGA TGA GTT GTA CC	
Swo1	forward	PVS159	CCC CCA AAC GGA ACA ACT TCC	
	rev	PVS160	CTG TAT CTG TGG TTG TGT AGG	
**CBM degenerated primers**				
**Box**	**orientation**	**primer**	**sequence**	**deg.**
GQCGG	Forward	FRG164	GGN CAR TGY GGN GG	64X
YSQC(L/I)	Reverse	FRG165	AD RCA YTG NGA RTA	96X
YSQC(L/I)	Reverse	FRG166	AD RCA YTG RCT RTA	32X
YAQC(L/I)	Reverse	FRG167	AD RCA YTG NGC RTA	96X
